# Acute Febrile Multisystem Illness With Vasculitic Features in a Young Adult: A Diagnostic Challenge Between Infectious and Autoimmune Etiologies

**DOI:** 10.7759/cureus.114791

**Published:** 2026-08-19

**Authors:** Tan Ho, Shreya Kansal, Amanda Sieverts, Michelle Lee, Kaitlin Huynh

**Affiliations:** 1 Internal Medicine, Valley Hospital Medical Center, Las Vegas, USA; 2 Internal Medicine, Touro University Nevada, Henderson, USA

**Keywords:** diagnostic uncertainty, febrile multisystem illness, palpable purpura, peripheral neuropathy, petechiae, rocky mountain spotted fever, tick-borne illness, transaminitis, vasculitis mimic

## Abstract

Acute febrile illness with rash and multisystem involvement in a young adult has a broad differential diagnosis spanning infectious and autoimmune etiologies. We report the case of a 21-year-old male who presented with fever, a mixed-morphology rash (petechiae and palpable purpura with centripetal spread), peripheral neuropathy, polyarthritis, and transaminitis following recent travel to Tennessee and Missouri. A prodromal cough two weeks prior to rash onset added diagnostic complexity.

Initial serologic testing during hospitalization was negative for Rocky Mountain spotted fever (RMSF) and dengue, and confirmatory convalescent serologies remained unavailable at discharge. Despite early corticosteroid administration for suspected small-vessel vasculitis, no clinical improvement was observed. Empiric doxycycline was initiated, given the epidemiologic risk for tick-borne illness.

This case highlights the critical importance of maintaining a broad differential, avoiding premature diagnostic closure, and prioritizing empiric antimicrobial therapy over early immunosuppression in diagnostically ambiguous, febrile multisystem illness.

## Introduction

Febrile multisystem illness presenting with rash is among the most diagnostically challenging syndromes encountered in internal medicine [1]. The overlap between tick-borne rickettsioses and primary autoimmune vasculitides can be striking; misdiagnosing these entities risks life-threatening outcomes from premature immunosuppression or delayed targeted antibiotics [2].

Rocky Mountain spotted fever (RMSF), caused by *Rickettsia rickettsii* and transmitted primarily by *Dermacentor variabilis* (American dog tick) and *Dermacentor andersoni* (Rocky Mountain wood tick), remains the most lethal tick-borne illness in the United States [3]. Despite its name, cases are concentrated in the South Atlantic and South Central states, including Missouri, Tennessee, Arkansas, and North Carolina [3]. Ehrlichiosis, transmitted by *Amblyomma americanum* (lone star tick), and anaplasmosis represent additional tick-borne entities capable of producing overlapping systemic features [3,4]. Critically, classic findings such as thrombocytopenia or a documented tick bite may be absent early in the disease course, and serology often lags clinical illness by one to two weeks, making early diagnostic confirmation difficult or impossible [3,5].

Meningococcemia is another hyperacute differential entity, characterized by the abrupt onset of fever and a petechial or purpuric rash. This can rapidly progress to purpura fulminans, severe hypotension, acute adrenal hemorrhage (Waterhouse-Friderichsen syndrome), and multiorgan failure. [6]. Hemorrhagic skin lesions are present in 28% to 77% of patients with invasive meningococcal disease upon admission, with median time from symptom onset to hospital admission as short as 12 to 13 hours in severe cases [6,7]. 

Autoimmune small-vessel vasculitides, particularly IgA vasculitis (formerly Henoch-Schönlein purpura), can similarly produce palpable purpura, arthralgias, and renal involvement [8]. However, prominent fever, pronounced leukocytosis, neuropathy, and hepatic enzyme abnormalities in adults are far more characteristic of systemic infection than primary vasculitis. This case explores the diagnostic tensions between these entities and offers a practical framework for approaching diagnostic uncertainty.

## Case presentation

A 21-year-old previously healthy male presented with a five-day history of acute febrile illness, diffuse myalgias, and progressive bilateral lower extremity pain and weakness. Notably, the patient reported a self-limited cough approximately two weeks prior to the onset of his current symptoms, which had since resolved without treatment. In the two weeks prior to presentation, he engaged in work-related travel to Tennessee and Missouri, though a detailed exposure history revealed predominantly indoor activity with minimal outdoor exposure.

**Figure 1:**
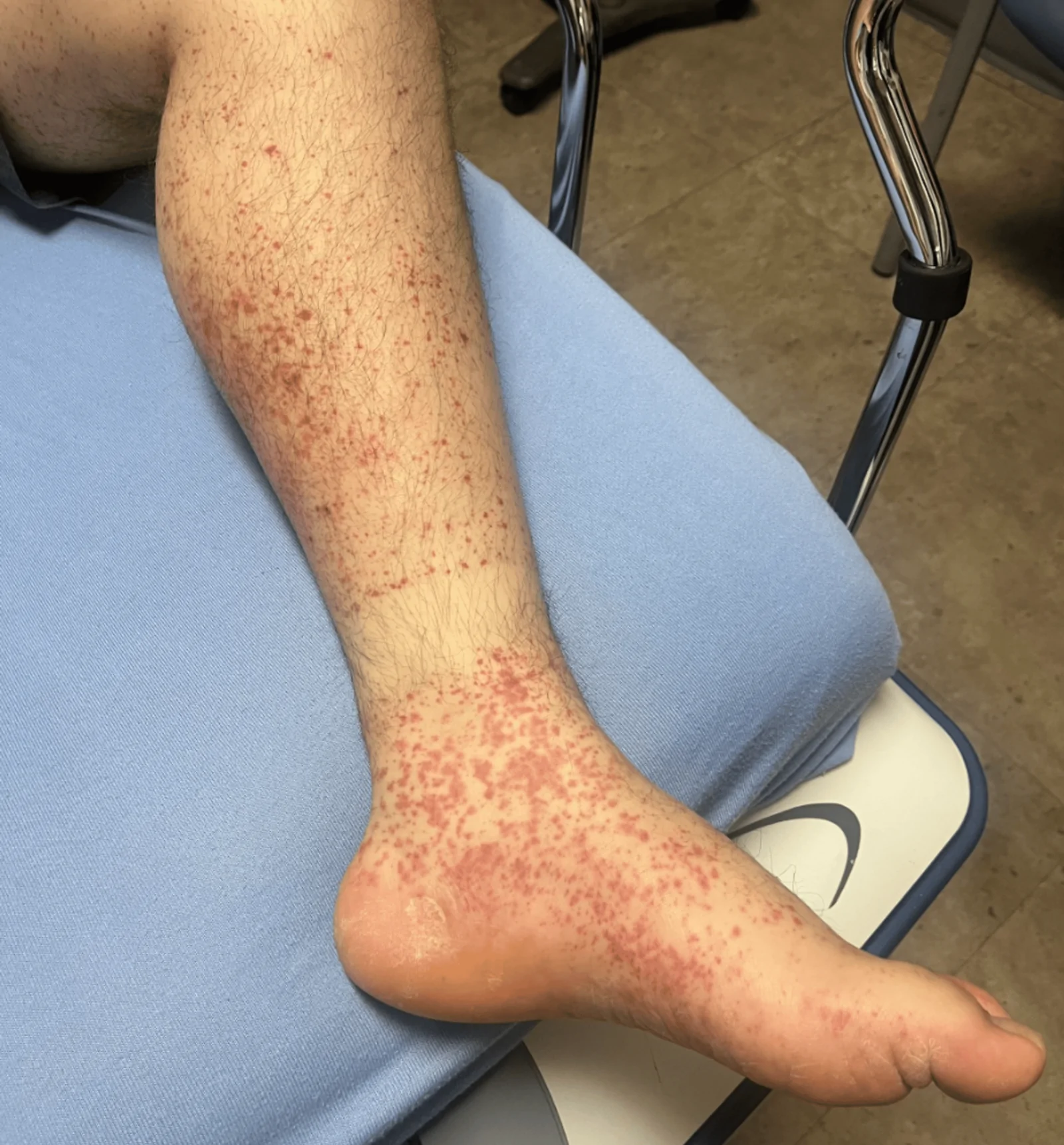
Macroscopic view of the petechial and purpuric rash involving the bilateral lower extremities, demonstrating involvement of the dorsum of the foot while sparing sock-banding pressure zones.

On initial presentation (Hospital Day 1), the patient was febrile at 39.1°C and tachycardic at 108 beats per minute. Physical examination was notable for a mixed-morphology rash involving the bilateral lower extremities, consisting of distal petechiae and evolving palpable purpura extending over the trunk. Of clinical significance, the rash distribution spared pressure-dependent regions, including areas corresponding to sock banding (Figure 1), a pattern atypical for gravitational-dependent vasculitic processes. The rash demonstrated a centripetal spread, originating at the distal extremities and advancing proximally and centrally, a pattern that strongly raises concern for rickettsial or systemic infection rather than primary cutaneous vasculitis.

Clinical evolution

By hospital day 4, the rash extended to involve the bilateral upper extremities, demonstrating centripetal evolution consistent with an active systemic process. Physical examination revealed a sensory deficit in a stocking-glove distribution and diminished distal deep tendon reflexes, consistent clinically with a peripheral neuropathy pattern. Electrodiagnostic studies, including nerve conduction studies and electromyography, were not performed during the acute hospitalization due to the patient's rapidly evolving systemic presentation and prompt clinical improvement following therapy. Musculoskeletal examination demonstrated diffuse tenderness and polyarticular joint swelling. Abdominal examination remained benign throughout hospitalization.

Hospital Day 1

Initial laboratory evaluation demonstrated marked leukocytosis (19.50 x 10³ μL) without initial thrombocytopenia (356 x 10³ μL), alongside mild transaminitis (aspartate aminotransferase (AST) 72 U/L, alanine transaminase (ALT) 72 U/L) and elevated alkaline phosphatase (196 U/L) (Table 1). Inflammatory markers were elevated (CRP >0.8 mg/dL, ferritin 453 μg/L). Lumbar puncture yielded normal cerebrospinal fluid (CSF) findings. Given the epidemiological risk for tick-borne rickettsiosis, empiric antimicrobial therapy was immediately initiated with oral doxycycline (100 mg twice daily) and intravenous ceftriaxone (2 g daily). 

Hospital Day 2

The patient developed worsening severe bilateral lower extremity hyperesthesia and severe deep aching pain. Due to persistent high fevers, progressive palpable purpura, and mounting concern for acute primary small-vessel vasculitis, immunosuppressive pulse-dose corticosteroid therapy was added with intravenous methylprednisolone (1000 mg IV daily for three consecutive days). 

Hospital Day 3

Transaminases peaked (AST 99 U/L, ALT 97 U/L) (Table 1). Neurologic examination demonstrated distal motor weakness and sensory deficits involving the hands and feet in a glove-and-stocking distribution, consistent with peripheral neuropathy. Musculoskeletal examination revealed diffuse tenderness and polyarticular joint swelling. Low complement component C4 was noted (2.7 mg/dL), with normal C3 (128.4 mg/dL). Acute serologies for RMSF (IgG/IgM) and dengue virus returned negative. 

**Table 1:** Serial laboratory values during hospitalization.

Laboratory Parameter	Day 1 (3/23)	Day 3 (3/25)	Day 4 (3/26)	Reference Range
White Blood Cells (K/µL)	19.50	20.13	16.35	4.5 - 11.0
Hemoglobin (g/dL)	13.2	12.4	12.9	13.8 - 17.2
Platelets (K/µL)	358	332	356	150 - 450
Aspartate Aminotransferase (U/L)	72	99	70	10 - 40
Alanine Aminotransferase (U/L)	72	97	123	7 - 56
Alkaline Phosphatase (U/L)	196	180	185	44 - 147
Total Bilirubin (mg/dL)	1.0	0.4	0.3	0.1 - 1.2
Serum Creatinine (mg/dL)	0.54	0.59	0.60	0.7 - 1.3
Ferritin (μg/L)	453	—	—	20 - 300
C-reactive Protein/Procalcitonin	Elevated	—	0.26	CRP: <0.8 mg/dL, Procalcitonin: <0.15 ng/mL
Rheumatoid Factor	—	—	6.7	< 14 IU/mL
C3/C4	—	128.4 / 2.7	—	C3: 90 – 180 mg/dL. C4: 14 – 40 mg/dL
Rocky Mountain Spotted Fever IgG/IgM	Negative	—	—	Negative (or Titer <1:64)
Dengue Serology	Negative	—	—	
Gonorrhea/Chlamydia Polymerase Chain Reaction	Not Detected	—	—	

Hospital Day 4

The rash extended proximally to involve the trunk and bilateral upper extremities, demonstrating dynamic, centripetal evolution. ALT peaked at 123 U/L while WBC count began to trend down (16.35 x 10³ μL) (Table 1). Despite completion of three consecutive daily pulses of high-dose IV methylprednisolone following initial doxycycline administration, no objective attenuation of rash progression, peripheral neuropathic symptoms, or inflammatory markers was observed. 

Laboratory findings

Serial laboratory evaluations were performed throughout the hospital stay to monitor disease progression and systemic organ involvement. These investigations revealed severe leukocytosis without initial thrombocytopenia, accompanied by marked elevations in liver transaminases and alkaline phosphatase (Table 1).

Notably, the pattern of leukocytosis without thrombocytopenia, combined with rising transaminases and alkaline phosphatase, is consistent with an inflammatory or infectious hepatic insult. The transient elevation in bilirubin, followed by a downtrend in the absence of reticulocytosis or hemoglobin decline, effectively excluded hemolytic processes. CSF analysis was unremarkable. Complement levels showed low complement C4 at 2.7 mg/dL (reference range: 14-40 mg/dL) with normal complement C3 at 128.4 mg/dL (reference range: 90-180 mg/dL). While this pattern can occur in autoimmune conditions or reflect inflammatory consumption, it is non-specific. Rheumatoid factor was unremarkable. Initial microbiological evaluation included serial blood cultures, which yielded no growth. Diagnostic testing for alternative infectious etiologies including viral exanthems (HIV, hepatitis B virus, hepatitis C virus, Epstein-Barr virus (EBV), cytomegalovirus (CMV), and parvovirus B19) was negative, making an active primary viral exanthem unlikely. Diagnostic imaging was unrevealing.

Diagnostic workup and serologic limitations

Serologic testing for RMSF (IgG and IgM) and dengue virus returned negative during the inpatient hospitalization. It is critical to recognize that RMSF serology has well-documented limitations in the acute phase of illness: IgM titers may not become detectable until seven to 14 days after symptom onset, and a single negative early titer should not be used to exclude the diagnosis. Centers for Disease Control and Prevention (CDC) guidelines explicitly state that empiric treatment should not await serologic confirmation. Additionally, confirmatory convalescent titers (obtained two to four weeks after illness onset) were not available because the patient was discharged before this window. The negative acute serology therefore does not rule out RMSF or other tick-borne rickettsioses. Other pending studies included evaluations for viral etiologies, *Ehrlichia*, *Anaplasma*, and broader autoimmune panels, all of which remained outstanding at discharge.

Treatment course

Given the patient’s epidemiologic risk profile (travel to a tick-endemic region) and clinical presentation concerning for tick-borne rickettsiosis, empiric doxycycline 100 mg orally twice daily was initiated immediately upon admission, along with intravenous ceftriaxone for broad-spectrum coverage. Two days later, due to persistent systemic symptoms and palpable purpura raising concern for acute small-vessel vasculitis, immunosuppressive therapy was added with intravenous methylprednisolone 1000 mg IV daily for three days. Despite three days of high-dose IV methylprednisolone, the patient demonstrated no clinical or inflammatory response, which retrospectively supported an infectious etiology over a primary autoimmune vasculitis. The patient was discharged with instructions to follow up for convalescent serologies, though these were not obtained before the case concluded. Attempts were made to contact the patient post-discharge for convalescent serologic testing; however, the patient was lost to follow-up.

## Discussion

This case illustrates a common diagnostic dilemma in general internal medicine: an acute febrile multisystem illness in a young patient. The clinical presentation bridged the divide between infectious and autoimmune etiologies. This case is instructive precisely because diagnostic certainty was never achieved. This is a situation that clinicians must navigate thoughtfully, using a defensible decision-making framework.

Several features of this presentation warrant careful analysis. First, the rash morphology and distribution were instructive but insufficient in isolation. The presence of both petechiae (non-palpable, suggesting thrombocytopenia or capillary leak) and palpable purpura (suggesting small vessel vasculitis or immune complex deposition) in the same patient reflects either simultaneous processes or disease evolution [2,3]. The centripetal spread, beginning at the distal extremities and advancing proximally, is a classic recognized pattern in RMSF [3,5]. This distribution is distinctly different from the predominantly lower-extremity, gravity-dependent presentation of adult IgA vasculitis [8]. The explicit sparing of sock-banding pressure zones further argues against a purely hydrostatic or mechanical purpura mechanism.

Second, the two-week prodromal cough prior to rash onset is an underappreciated feature that broadens the differential diagnosis. A respiratory prodrome preceding a systemic febrile illness with rash raises consideration of several viral etiologies, including parvovirus B19, EBV, and CMV [1]. Atypical bacterial pathogens such as *Mycoplasma pneumoniae* should also be considered, as these agents have all been associated with post-infectious, vasculitis-mimicking eruptions [8]. However, none of these were serologically confirmed during the acute inpatient stay.

Third, the absence of initial thrombocytopenia deserves explicit comment. While thrombocytopenia is classically associated with *Rickettsia rickettsii* and *Ehrlichia chaffeensis* infections, its absence early in the disease course does not exclude these diagnoses [1,5]. Several series have documented RMSF presenting with normal or near-normal platelet counts within the first several days of illness [5,9]. In ehrlichiosis, leukopenia and thrombocytopenia frequently develop later in the disease timeline [4]. The persistent leukocytosis observed in this patient is more consistent with an active bacterial or rickettsial insult than with a primary autoimmune process, in which leukocytosis is generally mild or non-existent [1,2].

Fourth, the pattern of elevated transaminases, elevated alkaline phosphatase, and transient hyperbilirubinemia is a characteristic feature of tick-borne rickettsioses [1,3,4]. This hepatic profile is distinctly atypical for primary IgA vasculitis in adults [8].

Fifth, the lack of clinical response to high-dose corticosteroids serves as an important retrospective clue. Intravenous methylprednisolone (1000 mg daily) was initiated on Hospital Day 2, 24 hours after starting empiric doxycycline, and was administered for three consecutive days. Although steroid non-responsiveness is not a formal diagnostic test, the absence of clinical improvement during or after this course provides indirect support for an infectious rather than a primary autoimmune etiology [2,8]. While initiating immunosuppression in the setting of an undifferentiated febrile illness was clinically understandable given the suspicion for vasculitis, it highlights the inherent risks of empirical immunosuppression before active infection, such as rickettsiosis, is excluded.

Finally, the laboratory limitations and diagnostic endpoints require balanced interpretation. Negative acute RMSF serology (IgG/IgM) during the first week of illness is expected, as detectable antibody titers often take 7 to 14 days to develop [1,10]. Both the CDC and the Infectious Diseases Society of America (IDSA) explicitly state that empiric doxycycline must be started immediately without awaiting serologic confirmation [1,10]. Multiple attempts were made to contact the patient post-discharge to obtain paired convalescent serologies; however, the patient was lost to follow-up. This single-case study has inherent limitations: without confirmatory paired titers, clinical recovery cannot be definitively attributed to doxycycline alone, and a self-limiting process cannot be completely excluded. Nevertheless, this case offers an instructive example of managing diagnostic ambiguity: favorable clinical outcomes can be achieved when sound epidemiologic reasoning and prompt empiric antimicrobial coverage take priority over premature diagnostic closure.

Key clinical takeaways emerge from this diagnostic dilemma. First, the early presentation of tick-borne rickettsioses can closely mimic primary autoimmune vasculitis, necessitating that empiric antimicrobial therapy take absolute priority over immunosuppression until infection is confidently ruled out. Second, clinicians must keep in mind the serologic and hematologic limitations of acute RMSF, as acute serology and early platelet counts are frequently normal during the first week of illness and must never delay empiric doxycycline. Lastly, physical pattern recognition, such as distinguishing centripetal from gravity-dependent rash distribution, alongside elevations in hepatic enzymes, provides crucial early clues that tilt the diagnostic balance toward rickettsial infection rather than autoimmune vasculitides.

## Conclusions

This case of an unresolved febrile multisystem illness in a 21-year-old with travel to tick-endemic regions offers a valuable opportunity for teaching the practical application of clinical reasoning under diagnostic uncertainty. The inability to confirm a final diagnosis is not a limitation of this report, but rather an instructive feature. Clinicians are frequently required to manage patients with undifferentiated illness; in suspected tick-borne disease, the imperative for prompt intervention is well established, as delaying antimicrobial therapy carries a significant risk of mortality.

Empiric doxycycline remains the treatment of choice for suspected rickettsial illness regardless of serologic confirmation status. Clinicians should resist the impulse to initiate immunosuppressive therapy based on rash morphology alone, and should integrate epidemiologic history, clinical trajectory, and laboratory trends into a dynamic, evolving differential diagnosis. When serology is negative or unavailable, convalescent testing should be planned prospectively at discharge. This case reinforces that the absence of diagnostic certainty is not uncommon in clinical medicine; sound empiric management, grounded in epidemiologic and clinical reasoning, can achieve favorable outcomes even without definitive confirmation.
